# Evaluating a large language model (ChatGPT-5) for detecting potential drug–drug interactions in intensive care: a cross-sectional comparative study with a clinical decision support system

**DOI:** 10.3389/fphar.2026.1911849

**Published:** 2026-09-21

**Authors:** İlkay Ceylan, Serpil Ekin, Buket Özyaprak, Derful Gülen

**Affiliations:** Department of Anesthesiology and Reanimation, Bursa Yüksek Ihtisas Training and Research Hospital, University of Health Sciences, Bursa, Türkiye

**Keywords:** artificial intelligence, clinical decision support systems, drug interactions, intensive care unit, pharmacovigilance

## Abstract

**Background/Objectives:**

Polypharmacy in intensive care units (ICUs) substantially increases the risk of potential drug–drug interactions (pDDIs), which may lead to adverse drug events and worsen clinical outcomes. Conventional rule-based clinical decision support systems (CDSSs), although widely used, are limited by static logic and restricted contextual reasoning. Recently, large language models (LLMs) such as ChatGPT have emerged as potential adjunctive tools capable of generating clinically interpretable outputs; however, their diagnostic reliability in safety-critical settings remains unclear.

**Methods:**

This cross-sectional study aimed to evaluate the diagnostic performance of ChatGPT-5 in detecting clinically significant pDDIs in ICU patients, using the UpToDate Drug Interaction Checker as the reference comparator. Data were collected from 101 adult ICU patients on a predefined index date. The model referred to as “ChatGPT-5” corresponds to the version available via the OpenAI API in September 2025. A standardized prompt was applied for each patient to ensure consistency. Diagnostic performance metrics, including sensitivity, specificity, positive predictive value (PPV), negative predictive value (NPV), overall accuracy, and Cohen’s κ coefficient, were calculated.

**Results:**

ChatGPT-5 identified a higher number of pDDIs per patient compared with UpToDate (median [IQR]: 6 vs. 0 [0–2]). The mean number of pDDIs per patient was also higher for ChatGPT-5 (7.2 ± 4.1) compared with UpToDate (1.1 ± 1.8). The diagnostic performance analysis was conducted at the patient level. Of the 101 included patients, 17 without a category D or X interaction in the UpToDate reference assessment were excluded from this analysis, leaving 84 patients. Sensitivity, specificity, and overall accuracy were 69.8%, 52.4%, and 65.5%, respectively, with a positive predictive value of 81.5% and negative predictive value of 36.7%. In a conservative full-cohort scenario analysis, treating the 17 excluded patients as discordant cases reduced overall exact agreement from 65.5% (55/84) to 54.5% (55/101). Agreement between the two systems was poor (κ = 0.19), indicating considerable variability in classification.

**Conclusion:**

Although ChatGPT-5 demonstrated limited diagnostic performance and the ability to generate clinically interpretable explanations, its low specificity and limited agreement with a rule-based system highlight important safety concerns. These findings suggest that LLMs may serve as complementary tools rather than standalone solutions in ICU pharmacovigilance. Further multicenter validation studies are required before clinical integration.

## 1 Introduction

Polypharmacy is a prevalent and complex issue in intensive care units (ICUs), where critically ill patients routinely receive multiple medications to manage comorbidities and acute conditions. This practice substantially increases the risk of potential drug–drug interactions (pDDIs), which can lead to adverse drug events (ADEs), increased morbidity, and prolonged ICU stays (Riera et al., 2022; Dagdelen et al., 2021). Reliable identification and classification of pDDIs are therefore essential components of medication safety in critical care settings (Bischof et al., 2025; Gottlieb and Silvis, 2023; Lu et al., 2023; Edwards et al., 2025; Liu et al., 2023; Gülen et al., 2023; Kim et al., 2025).

Clinical decision support systems (CDSSs), such as the UpToDate Drug Interaction Checker, are widely used to detect known pDDIs based on curated databases and deterministic rules. While these tools are well-established and clinically trusted, they often lack flexibility in handling contextual nuances or synthesizing unstructured inputs (Riera et al., 2022; Dagdelen et al., 2021). Additionally, rule-based systems may underreport interactions when drug combinations fall outside the predefined logic or database scope, potentially leading to missed alerts.

Recent advances in natural language processing (NLP) have introduced LLMs such as ChatGPT (developed by OpenAI) as potential adjunctive tools in healthcare. These models are capable of processing and generating complex text-based outputs, including clinical summaries and pharmacological reasoning. In early exploratory studies, ChatGPT has demonstrated an ability to identify and explain drug interactions, offering detailed narratives and mechanistic insights that may support clinician understanding (Park et al., 2024; Bischof et al., 2025; Gottlieb and Silvis, 2023). Despite these promising features, LLMs have not yet been validated for use in safety-critical environments such as ICUs. Concerns remain regarding their reliability, sensitivity to prompt phrasing, lack of source transparency, and the risk of generating inaccurate or “hallucinated” content (Lu et al., 2023; Edwards et al., 2025). Therefore, before considering any role for LLMs in clinical pharmacovigilance workflows, it is essential to benchmark their performance against established tools (Gottlieb and Silvis, 2023; Liu et al., 2023). ChatGPT does not have direct access to proprietary databases or real-time clinical resources. Instead, it generates responses based on patterns learned during training, which may include publicly available biomedical information and general knowledge about drug interactions. While it may reflect concepts found in sources such as DrugBank, Medscape, or published literature, it does not reliably replicate the structured logic of systems like Micromedex or UpToDate. As such, LLMs may offer a time-efficient and interpretive adjunct, but not a replacement, for validated tools in detecting pDDIs.

This study aims to explore the diagnostic characteristics of ChatGPT-5 in identifying pDDIs among ICU patients, using UpToDate’s Drug Interaction Checker. Rather than assessing ChatGPT for clinical deployment, the objective is to benchmark its detection profile, highlight discrepancies, and assess whether such a tool may eventually serve a complementary role alongside traditional systems. We hypothesized that ChatGPT-5 would demonstrate modest diagnostic performance and limited agreement compared with a structured rule-based CDSS.

## 2 Materials and methods

### 2.1 Study design and setting

This study was conducted after ethical approval by the Ethics Committee of University of Health Sciences Bursa Yüksek İhtisas Training and Research Hospital under the protocol number 2024-TBEK 2025/07-03 as a cross-sectional, point-prevalence analysis in the intensive care units of a teaching hospital. Written informed consent was obtained from all individual participants included in the study. For patients who were unable to provide consent due to impaired consciousness or medical incapacity, consent was obtained from their legally authorized first-degree relatives. Clinical trial number: This study is registered at ClinicalTrials.gov (Identifier: NCT07314125). The primary aim was to benchmark the performance characteristics of ChatGPT-5 in detecting pDDIs, using the UpToDate Drug Interaction Checker as the reference comparator. UpToDate is a subscription-based clinical decision support resource that was accessed through institutional hospital membership. Data were collected from ICU patients on a single predetermined day in September 2025, ensuring consistency in clinical and prescribing patterns. As the study involved retrospective data extraction and no deviation from standard care, it was classified as minimal risk under institutional review policies.

### 2.2 Study population

Eligible participants included adults aged 18 years or older who had been admitted to the ICU for at least 48 h and were receiving four or more concurrent systemic medications. Only patients with complete and accessible electronic medical records (EMRs), including detailed medication orders, were included in the analysis.

Patients were excluded if they had incomplete clinical or medication documentation, were receiving investigational or off-label therapies not represented in standard interaction databases, or were pediatric or pregnant individuals.

### 2.3 Data collection

Patient medication profiles were extracted directly from the hospital’s EMR system on the index day. An intensivist and a clinical pharmacist reviewed and verified all medication lists for accuracy and completeness. For each eligible patient, a final medication list was created to serve as the input for both interaction-checking systems.

### 2.4 Drug–drug interaction assessment tools

#### 2.4.1 UpToDate drug interaction checker

Used as a reference rule-based CDSS, UpToDate evaluates drug interactions using a structured evidence hierarchy, assigning severity levels ranging from “None” to “Contraindicated” (A–X). All interactions flagged by this tool were recorded along with their classification. To minimize false-positive alerts and align with ICU-tailored pDDI screening, clinically intentional duplicate or multi-agent regimens such as dual or triple antihypertensive or antiepileptic therapies were not considered potential interactions.

The UpToDate Drug Interaction Checker classifies interactions into categories B (no action needed), C (monitor therapy), D (consider therapy modification), and X (avoid combination), along with “None” indicating no known interaction.

#### 2.4.2 ChatGPT-5

ChatGPT-5 (OpenAI, accessed via API) was used to simulate a text-based clinical consultation. For each patient, a fixed prompt was submitted to standardize interaction detection and minimize variability:

“You are a clinical pharmacologist. I am an intensivist providing a list of my patient’s medications. Please identify any potential drug–drug interactions and report only those classified as major (D) or contraindicated (X).”

No additional role modifiers, system instructions, or clarifications were added. Medication lists were entered sequentially per patient. Outputs were recorded verbatim, including severity labels, pharmacological reasoning, and management suggestions where available. This setup was intentionally designed as a controlled benchmark scenario and not intended to reflect real-time clinical dialogue or full integration.

Artificial intelligence assistance was obtained using the commercially available GPT-5 product through the OpenAI API (OpenAI, San Francisco, CA, USA) between September 3 and 17 September 2025. The same API product designation, standardized prompt, and model configuration were used throughout the study period. The temperature parameter was fixed at 0 to minimize response variability. No additional system-level configuration or role-based instructions were used. The exact internal API model identifier and information regarding possible provider-side backend revisions were not accessible to the investigators; therefore, constancy of the underlying internal model version could not be independently verified.

All queries were performed using a standardized prompt to ensure consistency across patients. A separate conversation was initiated for each patient’s medication list, and each list was evaluated independently in a single-turn interaction using the same standardized prompt, without additional clarification or iterative refinement.

ChatGPT does not have real-time access to external clinical databases and generates responses based on patterns learned during training. Therefore, its outputs should be interpreted within the known limitations of generative AI systems.

The primary outcome of this study was the performance of ChatGPT-5 in detecting clinically significant pDDIs relative to the UpToDate Drug Interaction Checker, which was used as the reference comparator. Secondary outcomes included the number of pDDIs identified per patient and the concordance in severity classification between ChatGPT-5 and UpToDate. Additional measures included system agreement, assessed using Cohen’s κ coefficient, and diagnostic performance indicators such as sensitivity, specificity, positive predictive value (PPV), negative predictive value (NPV), and overall accuracy.

### 2.5 Statistical analysis

All statistical analyses were performed using IBM SPSS Statistics for macOS (version 30.0). Continuous variables were presented as mean ± standard deviation (SD) or median (interquartile range, IQR), depending on data distribution, which was assessed using the Shapiro–Wilk test. Non-normally distributed variables were summarized using median and IQR. Categorical variables were expressed as frequencies and percentages. The unit of analysis for the diagnostic performance and agreement analyses was the patient.

To evaluate diagnostic performance, a confusion matrix was constructed comparing ChatGPT-5 outputs with the UpToDate Drug Interaction Checker. For this analysis, only clinically significant interactions classified as category D (major) or X (contraindicated) by UpToDate were considered positive cases, while interactions classified as B, C, or “None” were considered negative. The analysis was restricted to patients with at least one category D or X interaction according to UpToDate because the primary objective was to evaluate the classification performance of ChatGPT-5 among patients with clinically significant interactions. Accordingly, 17 patients without a category D or X interaction in the reference assessment were excluded from the diagnostic performance analysis. To examine the potential influence of excluding these 17 patients without altering the primary D-versus-X classification analysis, a conservative full-cohort scenario analysis was conducted. Because UpToDate identified no category D or X interaction in these patients, whereas ChatGPT-5 generated at least one category D or X alert for each patient, all 17 patients were treated as discordant cases. The number of concordant classifications observed in the primary analysis was retained, and overall exact agreement was recalculated using the full cohort of 101 patients. Sensitivity, specificity, PPV, and NPV were not recalculated in this scenario because the added patients were not represented in the original D-versus-X contingency matrix.

For the primary D-versus-X classification analysis, category D was treated as the positive class and category X as the negative class. Discordant D/X classifications were categorized as false-positive or false-negative classifications according to the direction of disagreement. In cases of discordant severity classification between the two systems, only concordant D/X classifications between the two systems were considered positive agreement cases; all other mismatches were classified as false positives or false negatives as appropriate.

Sensitivity, specificity, positive predictive value (PPV), negative predictive value (NPV), and overall accuracy were calculated. Receiver operating characteristic (ROC) curve analysis was performed as an additional measure of discriminative capacity, expressed as the area under the curve (AUC). Agreement between ChatGPT-5 and UpToDate was assessed using Cohen’s kappa (κ) coefficient, interpreted according to the Landis and Koch scale.

## 3 Results

A total of 101 ICU patients were included, with a median age of 68 years (IQR: 54–76). Admission diagnoses were categorized into clinically relevant groups, with neurological disorders (28.7%) and respiratory failure/pulmonary diseases (27.7%) being the most common indications for ICU admission. Male patients comprised 63.4% (n = 64), and the median ICU stay was 12 days (IQR: 6–26). The median number of medications per patient was 8 (IQR: 4–16). The median number of pDDIs detected per patient was 0 (IQR: 0–2) for UpToDate and 6 (IQR: 4–10) for ChatGPT-5 (Table 1).

**TABLE 1:** Distribution of patients’ demographic and clinical characteristics (N = 101).

Variables	n (%)	Mean ± SD	Median (IQR)
Age (years)	​	64 ± 17	68 (54–76)
Gender			
Male	64 (63.4)	​	​
Female	37 (36.6)	​	​
Length of ICU stay (days)	​	20.4 ± 20.4	12 (6–26)
Admission diagnosis categories			
Respiratory failure/pulmonary diseases*	28 (27.7)	​	​
Neurological disorders^†^	29 (28.7)	​	​
Sepsis/infectious conditions^‡^	19 (18.8)	​	​
Trauma/postoperative/hemorrhagic conditions^§^	19 (18.8)	​	​
Metabolic/renal disorders^¶^	4 (4.0)	​	​
Cardiovascular/toxicologic conditions	2 (2.0)	​	​
APACHE II score	​	20.8 ± 7.8	20 (5–40)
Number of drugs	​	8.2 ± 2.7	8 (4–16)
Number of interactions (UpToDate)	​	1.1 ± 1.8	0 (0–2)
Number of interactions (ChatGPT-5)	​	7.2 ± 4.1	6 (4–10)

Abbreviations: ICU, intensive care unit; APACHE II, Acute Physiology and Chronic Health Evaluation II; IQR, interquartile range; SD, standard deviation.

* Respiratory failure/pulmonary diseases included pulmonary edema, aspiration pneumonia, hypercapnic respiratory failure, chronic obstructive pulmonary disease (COPD), pneumonia, and respiratory distress.

^†^ Neurological disorders included brain tumor, altered mental status, status epilepticus, subarachnoid hemorrhage, subdural hematoma, and stroke.

^‡^ Sepsis/infectious conditions included septic shock, meningitis, Fournier’s gangrene, and diabetic foot infection.

^§^ Trauma/postoperative/hemorrhagic conditions included traffic accident (multiple trauma), intra-abdominal hemorrhage, post-cardiopulmonary resuscitation (post-CPR) status, and postoperative TUR-M.

^¶^ Metabolic/renal disorders included acute renal failure, diabetic ketoacidosis, hyponatremia, and methanol intoxication.

### 3.1 Diagnostic performance

The diagnostic performance and agreement analyses were conducted at the patient level. Of the 101 patients included in the study, 17 without a category D or X interaction according to the UpToDate reference assessment were excluded from these analyses, leaving 84 patients. The D/X classification matrix showed 55 concordant classifications, including 44 D/D and 11 X/X agreements, and 29 discordant classifications, including 10 UpToDate-D/ChatGPT-X and 19 UpToDate-X/ChatGPT-D classifications (Figure 1).

**FIGURE 1:**
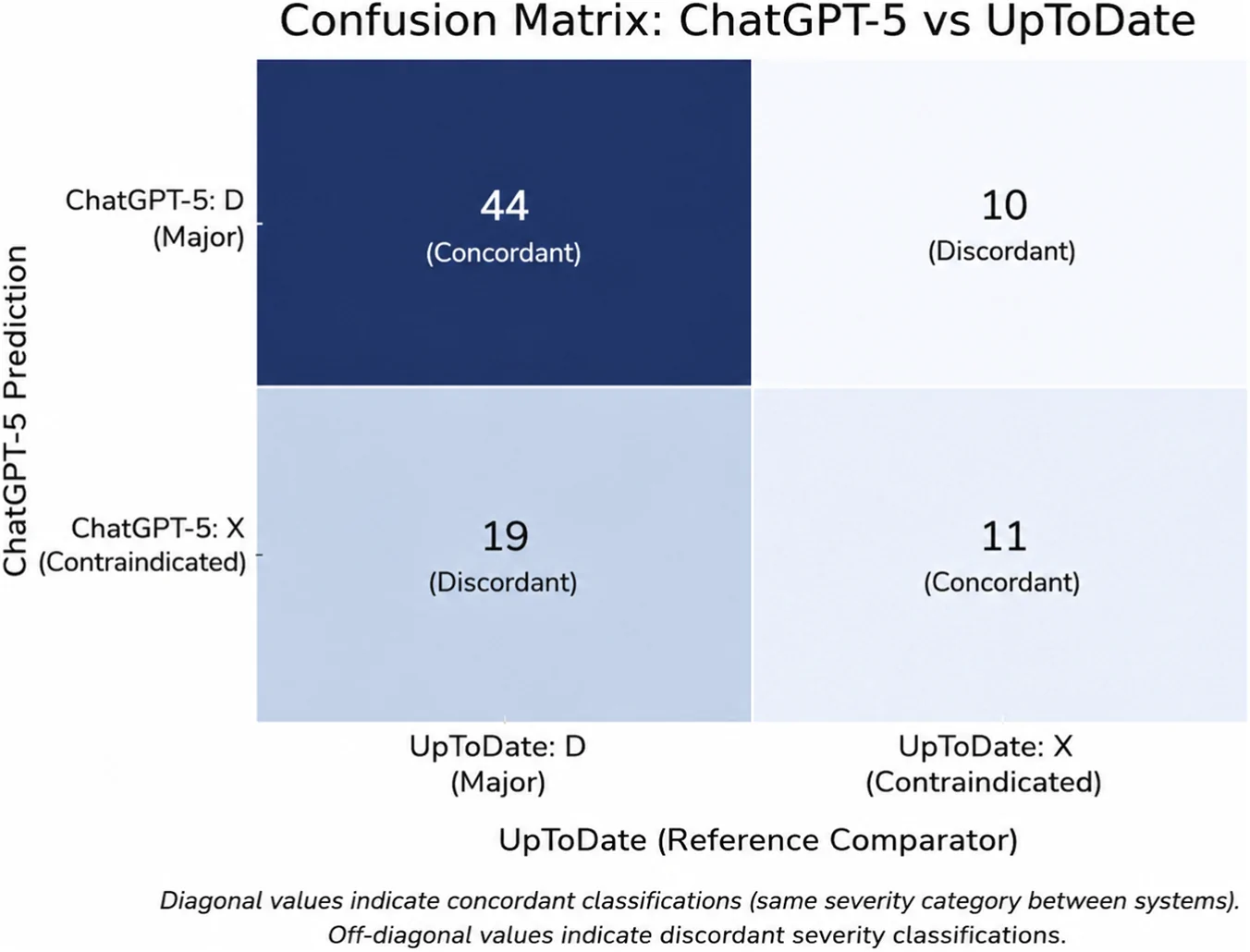
Confusion matrix illustrating agreement between ChatGPT-5 and UpToDate in the classification of clinically significant potential drug–drug interactions (pDDIs) as category D (major) or X (contraindicated). Diagonal values represent concordant classifications between the two systems, whereas off-diagonal values indicate discordant severity classifications.

ChatGPT-5 demonstrated a sensitivity (true positive rate) of 69.8% (95% CI: 57.4–80.1) and a specificity (true negative rate) of 52.4% (95% CI: 32.4–71.7). The PPV was 81.5% (95% CI: 68.6–90.7), the NPV was 36.7% (95% CI: 20.4–56.1), and the overall diagnostic accuracy of ChatGPT-5 in detecting pDDIs was 65.5% (95% CI: 54.3–75.3) (Table 2). ROC curve analysis yielded an AUC of 0.61, indicating limited discriminative performance (Figure 2).

**TABLE 2:** Patient-level diagnostic performance and D/X classification agreement between ChatGPT-5 and UpToDate among 84 patients.

Reference (UpToDate)/ChatGPT-5	D	X	Total
D	44	10	54
X	19	11	30
Total	63	21	84
Performance metrics			
Sensitivity	69.8% (95% CI: 57.4–80.1)	​	​
Specificity	52.4% (95% CI: 32.4–71.7)	​	​
PPV	81.5% (95% CI: 68.6–90.7)	​	​
NPV	36.7% (95% CI: 20.4–56.1)	​	​
Accuracy	65.5% (95% CI: 54.3–75.3)	​	​
Cohen’s κ	0.19	​	​
McNemar p	0.14	​	​

In a conservative full-cohort scenario analysis, the 17 patients without a category D or X interaction according to UpToDate were treated as discordant cases because ChatGPT-5 generated at least one category D or X alert for each patient. Overall exact agreement consequently decreased from 65.5% (55/84) to 54.5% (55/101). Other diagnostic performance metrics were not recalculated for this scenario because the additional patients were not represented in the original D-versus-X contingency matrix.

**FIGURE 2:**
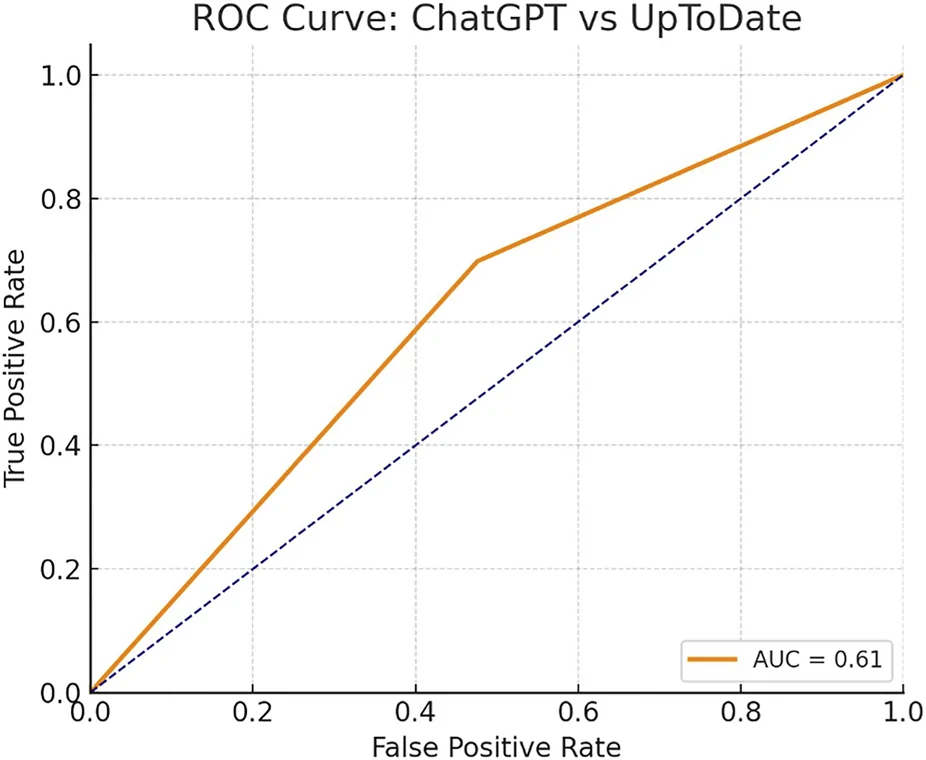
Receiver operating characteristic (ROC) curve illustrating the discriminative performance of ChatGPT-5 in detecting clinically significant potential drug–drug interactions (pDDIs), using UpToDate as the reference comparator. The area under the curve (AUC = 0.61) indicates limited discriminative ability.

### 3.2 Conservative full-cohort scenario analysis

In the primary restricted analysis, 55 of 84 patients had concordant D/X classifications. In a conservative full-cohort scenario analysis, the 17 excluded patients were added as discordant cases because UpToDate identified no category D or X interaction, whereas ChatGPT-5 generated at least one category D or X alert for each of these patients. Accordingly, overall exact agreement decreased from 65.5% (55/84) to 54.5% (55/101). Scenario-based sensitivity, specificity, PPV, and NPV estimates were not calculated because the additional patients were not part of the original D-versus-X classification matrix.

### 3.3 Agreement analysis

Cohen’s κ coefficient was calculated to assess the degree of agreement between the two systems. A κ value of 0.19 indicated poor agreement, according to Landis and Koch’s scale. Despite this, the McNemar test did not identify a statistically significant difference in the pattern of discordant cases between the systems (χ^2^ = 2.21, p = 0.14), suggesting that the distribution of classification errors was balanced and not systematically biased toward either method.

However, ChatGPT-5 did not identify 25 interactions classified as category D by UpToDate. Because UpToDate was used as a reference comparator rather than a definitive gold standard, these findings should be interpreted as clinically relevant discordances between the two systems.

### 3.4 Severity classification concordance

A notable and clinically significant finding was that ChatGPT-5 classified 323 out of 349 interactions (92.6%) identified as “no interaction” by UpToDate as major interactions (category D), indicating a substantial tendency toward overclassification. Table 3 presents the distribution of ChatGPT responses according to the interaction severity classes defined by UpToDate (B, C, D, X, or None). Of the 726 interaction alerts generated by ChatGPT-5, 639 (88.0%) were classified as category D and 87 (12.0%) as category X; both categories were considered clinically significant in this study (Figure 3).

**TABLE 3:** Interaction-level distribution of 726 ChatGPT-5 alerts according to UpToDate interaction categories.

UpToDate	ChatGPT D	ChatGPT X	Total
B	32 (91.4)	3 (8.6)	35 (100)
C	234 (89.3)	28 (10.7)	262 (100)
D	41 (69.5)	18 (30.5)	59 (100)
X	9 (42.9)	12 (57.1)	21 (100)
None	323 (92.6)	26 (7.4)	349 (100)
Total	639 (88)	87 (12)	726 (100)

**FIGURE 3:**
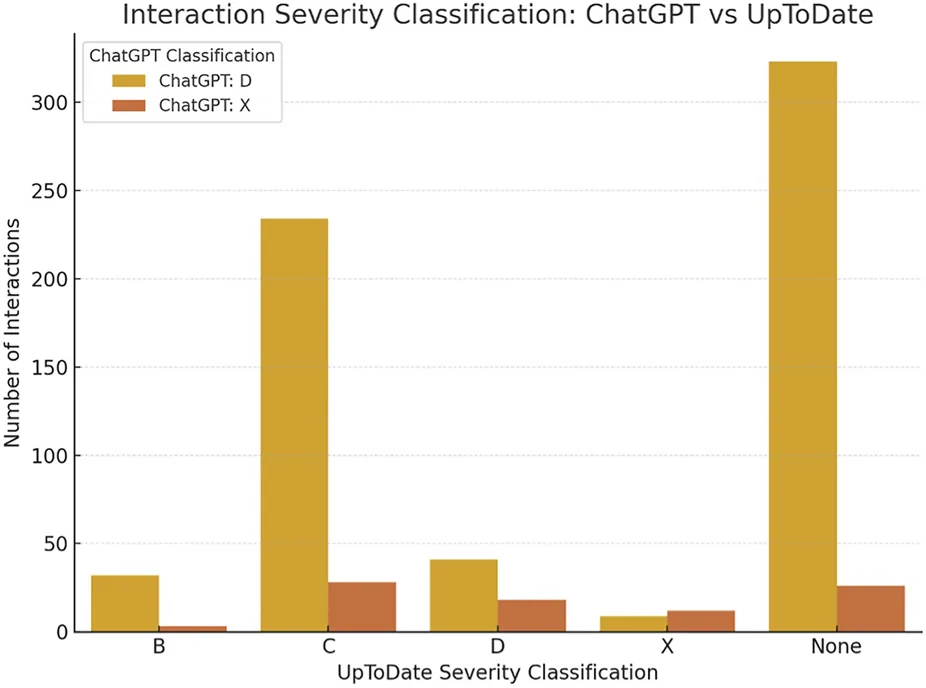
Interaction Severity Classification: ChatGPT-5 vs. UpToDate. Distribution of potential drug–drug interaction (pDDI) severity classifications as reported by UpToDate (categories B, C, D, X, and None) and ChatGPT-5. Across all severity levels, ChatGPT-5 predominantly classified interactions as “major” (D), including instances where UpToDate indicated no interaction. This pattern demonstrates relatively preserved sensitivity but low specificity, consistent with the earlier diagnostic performance metrics.

### 3.5 Conclusion of statistical findings

Although ChatGPT-5 generated more potential interaction alerts than UpToDate, the low κ value and suboptimal diagnostic accuracy indicate that it should not replace traditional CDSSs in critical care pharmacotherapy. Instead, ChatGPT may serve as a complementary resource, offering interpretive context or secondary validation, especially where traditional systems fall short in narrative reasoning or explainability (Figure 4).

**FIGURE 4:**
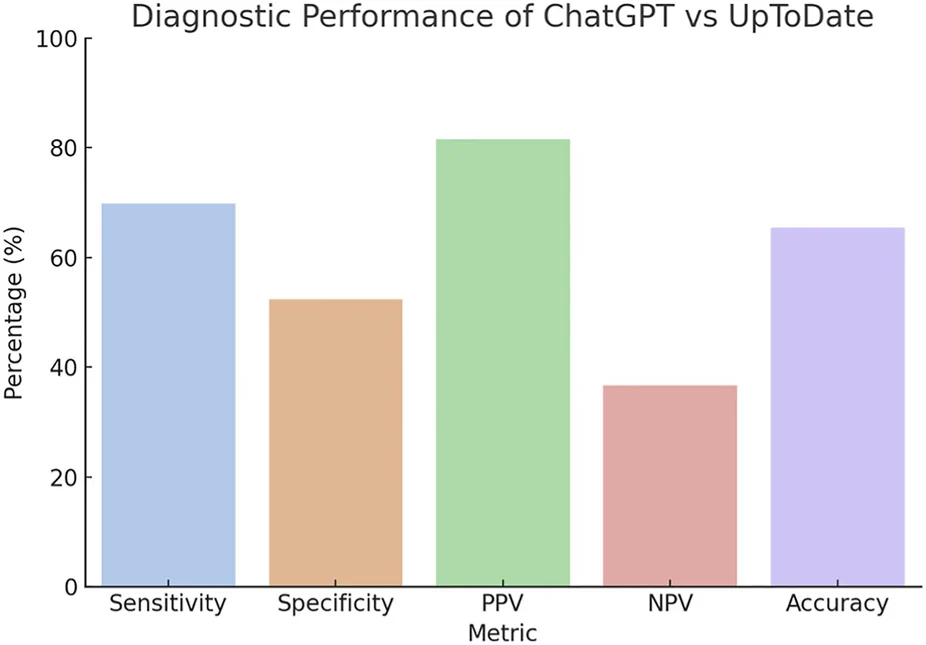
Diagnostic Performance Metrics: ChatGPT-5 vs. UpToDate. Bar chart summarizing the diagnostic performance of ChatGPT-5 compared with UpToDate in detecting potential drug–drug interactions (pDDIs). The metrics shown include sensitivity (69.8%), specificity (52.4%), positive predictive value–PPV (81.5%), negative predictive value–NPV (36.7%), and overall accuracy (65.5%). The distribution highlights ChatGPT-5’s relatively preserved sensitivity and high PPV, despite lower specificity and NPV, consistent with its tendency toward overclassification of interactions.

## 4 Discussion

This study evaluated the performance of ChatGPT-5, a large language model (LLM), in detecting pDDIs in critically ill ICU patients, using UpToDate as a rule-based comparator. The findings reveal that while ChatGPT identified a significantly greater number of interactions compared to the CDSS, this higher detection rate was accompanied by lower specificity and modest overall agreement, raising concerns regarding its suitability for safety-critical decision-making in its current form.

UpToDate was used as an established reference comparator rather than as a definitive gold standard. Although it is widely used in routine clinical practice, different drug-interaction databases may differ in database coverage, evidence appraisal, definitions of clinically relevant interactions, and severity-classification systems. Consequently, discordance between ChatGPT-5 and UpToDate does not necessarily establish that one system is definitively correct and the other incorrect. The findings should instead be interpreted as the performance and classification agreement of ChatGPT-5 relative to the selected comparator. Alternative reference approaches could include multidisciplinary expert adjudication or consensus across multiple drug-interaction databases, which may better account for differences in evidence appraisal and severity classification (Riera et al., 2022). However, this study was designed as a pragmatic benchmark against a single, widely used point-of-care clinical decision support resource. UpToDate was selected to provide one predefined and consistently applied classification framework across all patients. Nevertheless, the use of a single comparator limits the generalizability of the findings, and future studies should incorporate expert adjudication and/or multi-database consensus.

Previous studies conducted in Turkey have highlighted the high burden of pDDIs in ICU patients, reinforcing the critical need for efficient and accurate screening tools in local clinical environments (Gülen et al., 2023). Our findings align with these concerns by demonstrating a high interaction detection rate, though tempered by specificity limitations, suggesting that LLM-based systems may support—but not yet replace—traditional CDSSs in such high-risk settings (Gülen et al., 2023).

ChatGPT-5 exhibited a sensitivity (69.8%) and a strong positive predictive value (PPV: 81.5%), indicating that many of the flagged interactions were indeed clinically relevant. However, its low negative predictive value (36.7%) and poor agreement with the reference system (Cohen’s κ = 0.19) suggest a substantial rate of false negatives and limited reliability in excluding important interactions. Notably, the model failed to detect 25 interactions classified as high risk (category D) by UpToDate, which poses a potential safety hazard if relied upon exclusively in clinical workflows (Riera et al., 2022; Dagdelen et al., 2021).

The overprediction pattern observed—where ChatGPT tended to assign higher severity ratings, even in the absence of corroborating evidence—mirrors concerns raised in prior research on generative AI in medicine. Kim et al. (2025, preprint) specifically characterized such “medical hallucinations” in large foundation models, highlighting their potential to mislead clinicians through confident but unsupported outputs (Kim et al., 2025). These include risks of hallucination, over-alerting, and low precision in complex, high-stakes environments (Lu et al., 2023; Edwards et al., 2025). Such behavior could exacerbate alert fatigue, a well-documented barrier to effective CDSS adoption, especially in the ICU where clinicians are already overwhelmed with high-volume alerts (Liu et al., 2023; Bakker et al., 2024; Holbrook et al., 2025).

The high rate of overclassification observed in this study—where 92.6% of interactions labeled as “no interaction” by UpToDate were classified as major by ChatGPT—has important clinical implications. In high-acuity environments such as the ICU, excessive alert generation may contribute to alert fatigue, a well-recognized phenomenon in which clinicians become desensitized to frequent warnings. This may paradoxically increase the risk of overlooking truly critical drug interactions, thereby compromising patient safety.

One potential explanation for this overprediction behavior is the lack of patient-specific clinical context in the model input. ChatGPT evaluated drug interactions based solely on medication lists without access to key clinical parameters such as renal function, hepatic status, or comorbid conditions. As a result, pharmacokinetic and pharmacodynamic interactions may have been identified in a context-independent manner, leading to inflated severity classification and a high rate of false positives.

Nonetheless, ChatGPT’s capacity to generate narrative justifications for interactions, including mechanistic reasoning and contextual detail, may offer a valuable adjunct to traditional systems (Graafsma et al., 2024). This interpretive layer has been highlighted in earlier work as a possible strength of LLMs, particularly in settings involving polypharmacy or ambiguous clinical situations where a rule-based system may provide limited guidance (Park et al., 2024; Bischof et al., 2025). Studies like those by Edwards et al. have emphasized the role of LLMs in augmenting clinician understanding, rather than replacing clinical judgment or established tools outright (Edwards et al., 2025).

Bischof et al. (2025) similarly explored the use of ChatGPT for pDDI analysis but found that it detected substantially fewer interactions than conventional CDSSs and failed to identify key risks such as QTc prolongation. In their study, ChatGPT identified only 80 interactions compared to 280 identified by CDSSs, and inconsistencies were observed in 90% of repeated prompts, highlighting serious reproducibility issues.

A recent study by Tayeb et al. directly compared five large language models, including ChatGPT-5, with a panel of experienced oral surgeons in 500 standardized oral-surgery cases involving clinically relevant pDDIs. ChatGPT-5 demonstrated the highest sensitivity for actionable category D or X interactions (98.0%) but had substantially lower specificity (56.7%) and generated more false-positive warnings (Tayeb et al., 2025). Although that study was conducted in a dental setting and used expert consensus with a Lexicomp-based severity scale, its findings show a pattern consistent with the overclassification observed in our ICU population. Together, these findings suggest that high sensitivity may be accompanied by excessive alert generation and reinforce the need for human supervision when large language models are used for medication-safety screening.

In contrast, our study used a standardized fixed prompt across a larger cohort (n = 101), which minimized variability and allowed for a more reproducible performance assessment. Furthermore, while Bischof et al. included multiple CDSS comparators with differing logic structures, our focused 1:1 comparison with UpToDate enabled clearer benchmarking. However, the specific version of ChatGPT used in that study was not clearly reported, which may limit the direct comparability of results given the rapid evolution of model capabilities. These differences in design strengthen the reliability and interpretability of our findings in a critical care context. Similarly, Domian et al. (2025) compared the performance of AI-based platforms and DDI screening databases using real-world patient data, highlighting methodological gaps in existing tools—such as inconsistent data sources, lack of standardized severity classification, limited validation on real-world datasets, and inadequate incorporation of clinical context (Domián et al., 2025). Recent studies have also explored the use of ChatGPT in detecting drug–drug interactions, demonstrating variable performance depending on clinical context and methodology (Thapa et al., 2025).

The discordance between ChatGPT-5 and UpToDate also reflects fundamental architectural differences. While CDSSs like UpToDate rely on structured databases with transparent evidence grading, ChatGPT generates responses based on probabilistic pattern matching across a vast corpus of general text, which lacks traceable sources or context-specific tuning. This makes the model vulnerable to prompt variability, as slight changes in input phrasing can lead to different outputs—an issue not present in structured systems (Lu et al., 2023; Liu et al., 2023).

Importantly, the findings of this study are consistent with the emerging consensus in the literature: LLMs such as ChatGPT show potential as complementary decision support tools, especially for enhancing interpretability or filling knowledge gaps. However, they must be deployed with strict oversight, ideally in hybrid frameworks where AI-generated outputs are filtered, verified, and contextualized by traditional systems and clinician input (Bischof et al., 2025; Edwards et al., 2025). Similar to structured AI tools like AI-Antidelirium, real-world CDSS implementations require alignment with clinical workflows to improve adherence without adding cognitive burden (Zhang et al., 2025; Farhaoui et al., 2025; Corsello et al., 2025).

Our findings, unlike those of Bischof et al., suggest that under tightly controlled conditions with standardized prompts, ChatGPT-5 may offer value as a supplementary screening tool in high-risk environments such as the ICU. However, due to the ongoing limitations in sensitivity and specificity, its use should remain adjunctive and fully supervised until more consistent performance and clinical validation are achieved (Al-Ashwal et al., 2023).

From a clinical perspective, the findings of this study highlight important safety considerations. The relatively preserved sensitivity of ChatGPT-5 indicates that some clinically significant pDDIs may still be missed, which could lead to potential adverse events if the model is used as a standalone tool. At the same time, its tendency to over identify interactions and assign higher severity may contribute to increased alert burden and reduced clinical trust. Therefore, the use of ChatGPT-5 in ICU pharmacotherapy should remain strictly supervised and integrated within existing clinical decision support frameworks. Its most appropriate role may be as a complementary tool that enhances interpretive understanding rather than as an independent decision-making system.

The divergence in severity assessment further highlights the variability in interpretation between a rule-based system and a generative AI model, underscoring the need for human oversight in high-risk clinical scenarios.

### 4.1 Limitations

#### 4.1.1 Study design and population

The cross-sectional, single-center design limits the generalizability of the findings. In addition, the primary diagnostic performance analysis excluded 17 patients without a category D or X interaction according to UpToDate, resulting in a subgroup enriched for reference-positive patients and reducing the representation of patients with only category B, category C, or no interactions. In a conservative full-cohort scenario analysis, treating all 17 excluded patients as discordant cases reduced overall exact agreement from 65.5% (55/84) to 54.5% (55/101). Sensitivity, specificity, PPV, and NPV were not recalculated for this scenario because the original restricted matrix compared D and X severity classifications and did not include a separate reference-negative category. These findings nevertheless indicate that exclusion of the 17 patients resulted in a more favorable estimate of overall agreement. Accordingly, the primary performance estimates should be interpreted as conditional estimates for the selected subgroup and should not be generalized directly to an unselected ICU population. These findings indicate that the restricted analysis produced more favorable estimates of specificity, PPV, and overall accuracy. Therefore, the primary performance estimates should be interpreted as conditional estimates for the selected subgroup and should not be generalized directly to an unselected ICU population.

#### 4.1.2 Reference framework

UpToDate was used as an established reference comparator reflecting routine clinical practice rather than as a definitive gold standard. The study did not include multidisciplinary expert adjudication or consensus across multiple drug-interaction databases. In addition, formal inter-rater reliability was not assessed for the interpretation of UpToDate classifications or ChatGPT-5 outputs. Because interaction databases may differ in coverage, evidence appraisal, and severity classification, the observed performance estimates and discordances may vary when a different reference framework is used.

#### 4.1.3 Prompt and model configuration

ChatGPT-5 was evaluated using a fixed standardized prompt, a temperature setting of 0, and separate single-turn conversations for individual patient medication lists. Although this configuration improved procedural consistency, it may not reflect real-world use involving variable prompts, iterative clarification, or multi-turn clinical dialogue. Furthermore, the exact internal API model identifier and information regarding possible provider-side backend revisions were not accessible to the investigators; therefore, constancy of the underlying internal model version could not be independently verified.

#### 4.1.4 Clinical context and outcome data

The model received medication lists without patient-specific variables such as renal function, hepatic function, body weight, drug doses, or comorbidities. These factors may influence the clinical relevance and severity of pDDIs and may have contributed to the observed discordance. No clinical outcomes, including adverse drug events or ICU length of stay, were linked to the identified interactions; therefore, direct conclusions regarding clinical impact cannot be drawn. Because the study was designed as a diagnostic benchmarking analysis rather than a predictive modeling study, multivariable adjustment was not performed. Future multicenter prospective studies should incorporate patient-specific clinical data, clinical outcomes, expert adjudication, and multiple reference databases.

## 5 Conclusion

From an operational standpoint, integration of ChatGPT or similar LLMs into ICU pharmacotherapy workflows would require robust safeguards, including clear boundaries on autonomous use, real-time clinician validation, traceability of recommendations, and mechanisms to detect and suppress low-confidence or hallucinated content.

Future research should focus on multicenter, prospective study designs that integrate clinical outcomes, such as adverse drug events and ICU length of stay, to better evaluate the real-world impact of AI-assisted pDDI detection. Additionally, hybrid approaches that combine the structured reliability of rule-based CDSSs with the interpretive and explanatory capabilities of large language models may offer a more balanced solution, improving both diagnostic accuracy and clinical usability.

## Data Availability

The raw data supporting the conclusions of this article will be made available by the authors, without undue reservation.
